# Visualization Analysis of Defibration and Distributive Uniformity of Fibers in Long-fiber-reinforced, Injection-Molded Resins

**DOI:** 10.3390/polym12030727

**Published:** 2020-03-24

**Authors:** Sai Ma, Xiaobin Wu, Shigeru Owada, Hidetoshi Yokoi

**Affiliations:** Institute of Industrial Science, The University of Tokyo, Tokyo 153-8505, Japan; bin@iis.u-tokyo.ac.jp (X.W.); showada7@iis.u-tokyo.ac.jp (S.O.); hiyokoi@yokoi-labo.jp (H.Y.)

**Keywords:** injection molding, plastication, distribution, long-fiber-reinforced resins

## Abstract

The pursuit of mechanical strength in the injection molding of long-fiber-reinforced resins continues to pose major challenges, namely (1) improvement of fiber defibration and fiber distribution and (2) suppression of fiber breakage during the molding machine’s plastication process. In the present study, a new defibration and distribution evaluation mold is developed to quantitatively evaluate the defibration and distribution of long fibers in nozzle-injected resin. A quantitative analysis method using this evaluation mold is proposed for visualizing and observing long-glass-fiber-reinforced resin up to 30 wt % and long carbon fiber-reinforced resin up to 10 wt %. The method, based on the intensity of light transmitted from a backlight source, is also used to evaluate areas of undefibrated fiber pilling and for evaluating the influence of molding conditions on fiber defibration and uniform distribution. The results clarify that fiber distribution non-uniformity can be reduced by improving the concentration adjustment procedure for the dry blending of high-concentration pellets. Additional results show that fiber defibration and distributive uniformity can be improved by applying high back pressure.

## 1. Introduction

Using resin in place of metal parts to reduce weight in the context of molding is a key technological trend in the transportation equipment industry, particularly the manufacture of automobile and airplane parts. The application of carbon fiber and other long-fiber-reinforced resins to molding is, therefore, increasing. However, fiber breakage and fiber pilling due to incomplete fiber defibration are problems encountered when long-fiber-reinforced resin is injected in an injection molding context. These problems occur during resin material plastication in the heating cylinder [1,2,3,4,5,6,7] and when the resin passes through the narrow flow path of the nozzle through the reciprocating screw’s check-ring [8,9,10,11,12]. When evaluating the strength of injection-molded thermoplastic fiber-reinforced resin, high average fiber length does not always lend the molded product high mechanical strength. Uniform fiber distribution also contributes crucially to the molded product’s strength. The molded product’s accuracy and mechanical strength suffer when the cavity is filled with resins of non-uniform fiber distribution or consists of broken fibers. These deficiencies also cause poor appearance.

The fiber-reinforced resin gradually melts as it defibrates while being transferred from the hopper to the cylinder. In order to improve fiber distribution uniformity, material properties, and/or material production, processes must be improved, e.g., a screw with a dispersing function, such as a mixing element. For example, Kobe Steel Co., Ltd. developed an atmospheric pressure impregnation method that contains a high-concentration reinforcing material to improve the fibers’ distributive uniformity by allowing them to be dispersed one by one [13]. Mizuno et al. proposed a fractal dimension as a metric for evaluating distributive uniformity [14]. In their study, cross-sections of the sample were cut, and microscope images of the sections were binarized. Okada et al. analyzed the distributive uniformity of cellulose nanofibers in polypropylene using Fourier-transform infrared spectroscopy (FT–IR) images, leveraging the inherent scale-dependence of uniformity observations [15]. Terada et al. analyzed carbon fiber reinforced polymer (CFRP) microscopic images using a similar technique [16]. However, these static measurement evaluation methods use molded samples and are, therefore, unable to evaluate the defibration and distribution of non-uniform flowing into the mold during the molding process. Since only the dispersibility obtained from the observation of the cross-section is evaluated, the pilling phenomenon of the fiber and the dispersibility of the entire sample can rarely be evaluated in principle. Furthermore, the many definitions and quantification methods of defibration have not been established properly, making it difficult to evaluate the influence of distributive uniformity on the mechanical strength of molded products consistently or quantitatively.

To summarize the problems of past studies on fiber defibration and distributive uniformity, (1) most studies focused on fiber length, but few studies focused on fiber defibration and distributive uniformity. (2) The correlation analysis between fiber defibration and distributive uniformity and molding conditions has not been performed, and (3) there has been no visualization analysis of fiber-reinforced resins other than glass fibers. In the future, to improve the optimal product design and the productivity of non-defective products, it is increasingly necessary to establish an effective method for evaluating the defibration and distributive uniformity of fiber-reinforced resins. Furthermore, it is necessary to study glass fibers and carbon fibers. As described above, the promotion of visualization analysis of defibration and distributive uniformity of long carbon fiber and long glass fiber reinforced resin has currently become a critical research topic. For this reason, we herein propose a quantitative method to evaluate the distributive uniformity of long-fiber-reinforced resin extruded from a nozzle and injected into a mold in a reciprocating-screw plastication process. Our defibration and distribution evaluation mold provides a systematic evaluation basis for the actual defibration and distributive uniformity of the resin material obtained under various plastication conditions (especially the effect of backpressure conditions). Using the evaluation mold, we measure the intensity of light transmitted from a backlight source as a quantitative means of visualizing the defibration and distribution conditions of nozzle-injected long carbon fibers and long glass fibers in the molten resin.

## 2. Experimental Equipment, Conditions, and Method

### 2.1. Development and Observation Principle of Distributive Uniformity Evaluation Mold

**Figure 1:**
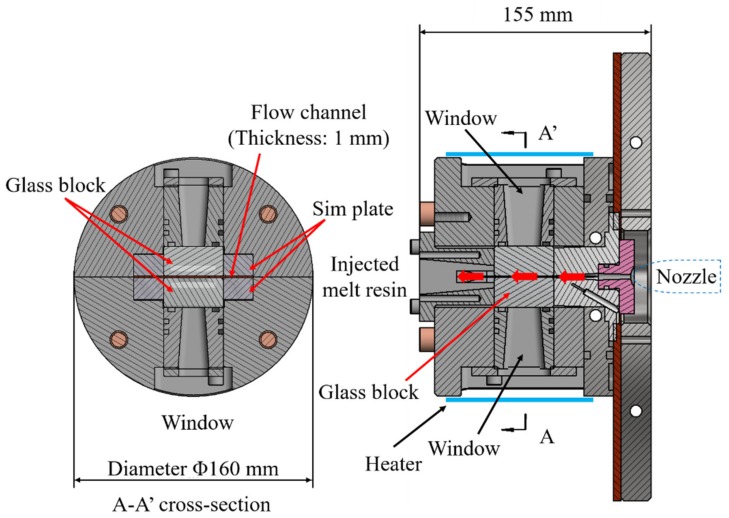
Fundamental structure of the newly developed evaluation mold.

**Figure 2:**
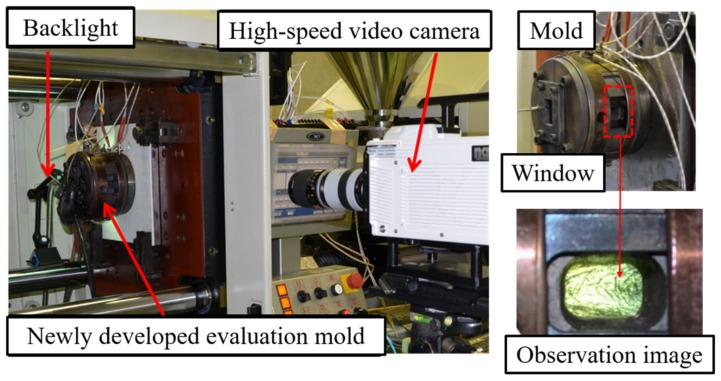
Evaluation mold mounted on the stationary side of platen.

The distributive uniformity evaluation mold developed in this study was based on coloring masterbatch mixability evaluation technology and consisted of a conventional glass insert mixing evaluation mold [17]. Figure 1 and Figure 2 show the basic structure and appearance of the distributive uniformity evaluation mold. The mold consisted of two halved steel cylindrical blocks (outer diameter 160 mm). A pair of quartz glass blocks (20 mm × 40 mm × 40 mm) was placed inside the cylindrical block, facing each other. A 1 mm spacer sheet was sandwiched between the glass blocks to form a channel shape of certain thickness. The flow thickness was set to 1 mm, and windows were installed opposite both glass blocks on the outside. Backlight transmission images, illuminated by high-intensity light from the back side, could then be taken by the high-speed video camera in front. Where the flow path shape transitioned from the nozzle (circular flow path) to the rectangular flat flow path in the glass area, a fan-shaped flow path was inserted so that undefibrated fibers would not remain. This setup eliminated micro steps and aligned the center of the flat flow path with the nozzle. In addition, to keep the entire resin flow path temperature above 200 °C, two Φ4 mm small heaters were also inserted around the exit after the resin material passed through the fan gate and visualization area. The cylindrical block was fixed to the mounting plate via a heat-insulating plate. Each part was sealed with an O-ring to prevent resin leakage into the visualization area. This detailed design realizes high resistance to pressure and high sealability for long-fiber-reinforced resin while permitting continuous visualization and observation of the defibration, and distributive uniformity of the fiber, in the resin injected via the nozzle.

### 2.2. Experimental Conditions and Method

**Figure 3:**
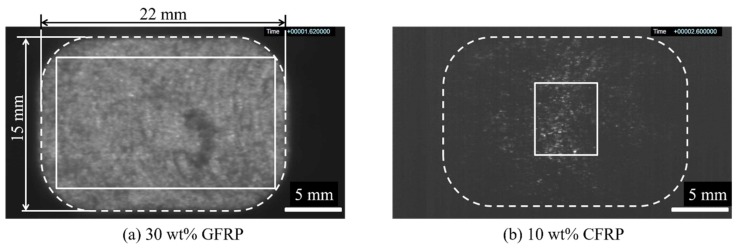
Observation image (Evaluation area: (**a**) 500 × 840 px (glass fiber reinforced polymer, GFRP); (**b**) 480 × 420 px (carbon fiber reinforced polymer, CFRP).

This experiment used a NEX110-18EPI electric injection molding machine (Nissei Plastic Industrial Co., Ltd., Nagano Prefecture, Japan; full-flight screw diameter 36 mm), MEMRECAM HX-3 high-speed video camera (NAC Image Technology Inc., Tokyo, Japan), and fiber light (Ushio Lighting Co., Ltd., Tokyo, Japan) as a light source for experiment visualization. Image J (NIH Image) was used as the image analysis software [18]. Experimental materials included long-glass-fiber-reinforced PP (Prime Polymer Co., Ltd., Tokyo, Japan; L-5050P; fiber content 50 wt %, fiber diameter 16 μm; fiber length 8 mm) and long-carbon-fiber-reinforced PP (Toray Industries, Inc., Tokyo, Japan; TLP8148, fiber content 20 wt %, fiber diameter 7 μm, fiber length 6.7 mm), each of which was dry blended and diluted with natural PP (Prime Polymer Co., Ltd., Tokyo, Japan; J137G). In the experiment in Section 3.1, 50 wt % of glass long fiber reinforced PP was diluted with natural PP to obtain 30 wt % of glass fiber reinforced resin. In the experiment in Section 3.2, 20 wt % carbon long fiber reinforced PP was diluted with natural PP to obtain 10 wt % carbon fiber reinforced resin. In the experiment in Section 3.3, 20 wt % of carbon long fiber reinforced PP was diluted with natural PP to obtain 5 wt % of carbon fiber reinforced resin. Table 1 shows the molding conditions, where the screw utilized is a conventional full-flight screw without any mixing zones, and the plastication conditions are not optimized for these resins. In this experiment, the effectiveness of the fiber dispersion evaluation method proposed below is examined by changing the backpressure level as a molding condition that should have the greatest effect on fiber dispersion. Figure 3 shows an example observed image with increased brightness after shooting. The whole image was taken at 1280 × 720 px and 2000 fps. In the entire image, the area to be analyzed was first measured to determine the velocity distribution in the image, and the velocity gradient areas near the upper and lower ends of the image were deleted. Furthermore, a rectangular area was formed by deleting low-luminance areas generated in the peripheral portion of the backlight image. For both the 5 wt % long-carbon-fiber resin and 30 wt % long-glass-fiber resin, a central rectangular area, measuring 500 × 840 px (length 11.6 × width 19.5 mm), was set as the evaluation area within each image. For the 10 wt % long-carbon-fiber resin, a central rectangular area (with strong backlight luminance), measuring 480 × 420 px (length 11.1 × width 9.7 mm), was analyzed. In addition, among the measurement images in each shot, the rising period, from the start of injection until the flow velocity became constant, was deleted from the evaluation target, leaving only images with stable velocity and pressure areas to be evaluated.

## 3. Experimental Results and Discussion

Long-fiber-reinforced resin became defibrated while passing through the heating cylinder’s screw channel. At that time, distributive uniformity was poor due to imperfect fiber defibration. When melted resin material containing fiber bundles with poor defibration and non-uniform distribution were injected via the nozzle and flowed through the narrow flow path between the glass blocks, brightness gradation was generated within the backlight image. There are two methods for evaluating the fiber bundles’ defibration and distributive uniformity based on the image darkness. One method extracts and analyzes the dark areas in the measured image, while the other method extracts and analyzes the bright areas. The former was used when the measurement image’s overall brightness was high, while the latter was used when the overall brightness was low. Below, each analysis method is explained in order, including each method’s effectiveness as clarified through specific application examples and analysis results. Finally, the two methods’ correlated results are discussed.

### 3.1. Dark Area Analysis Method

Long glass fiber is a transparent material, but its refractive index differs from that of transparent molten resin. Therefore, in areas with large quantities of fibers, light is difficult to transmit, and these areas appear dark in the backlight image. Distributive uniformity evaluation can be performed based on some kind of threshold value by extracting fiber areas (pilling areas) with poor distributive uniformity where fiber bundles are not defibrated.

Long carbon fiber is an opaque material. When the content is low, the average luminance of the backlit image is high, and the pilling area can be extracted as a dark area, as with long glass fiber. In addition, the matrix resin PP is generally opaque. When resin melts, the crystals in the resin disappear, and the resin becomes completely transparent, allowing the undefibrated area to be extracted by the camera easily.

The image’s dark area was determined by a grayscale value histogram. The ratio of the dark area to the total evaluation area was obtained, and the changes occurring during injection were evaluated. To evaluate the changes, first the average grayscale value (*I*_ave_: 256-tone grayscale value) of the evaluation area (*S*) was obtained from the video image of each injected shot. In each image, a dark pixel area (*P*), equal to or less than a pixel grayscale value (*L*) threshold (*Thres*), was obtained from the grayscale value histogram of the evaluation area and counted as a pilling area. Furthermore, the occupation ratio of dark pixels in the evaluation area was defined as the pilling ratio (*α*), calculated as

(1) $$\alpha =\sum_{j}^{area}P_{j(L_{j}<Thres)}/S$$

where *S* is the evaluation area, *P* is the pixel, *L* is the pixel grayscale value (256 grayscale values), *Thres* is the threshold value, and *area* is the total number of pixels in the image.

As described above, the dark area analysis method can be applied to visualization experiments using long glass fibers, as well as long carbon fibers, at low carbon content. In the following analysis, this method was used on 30 wt % glass-fiber-reinforced resin, and evaluation experiments investigated the influence of back pressure on the fibers’ distributive uniformity. The sampling interval of the images was set to 40 ms so that the moving distance, based on the maximum flow velocity *V*_max_, was equivalent to the flow length of 19.5 mm in the evaluation area. The evaluation area *S* in this experiment was 840 × 500 px.

#### 3.1.1. Changes in Average Brightness with Time during Resin Flow at Each Back Pressure

**Figure 4:**
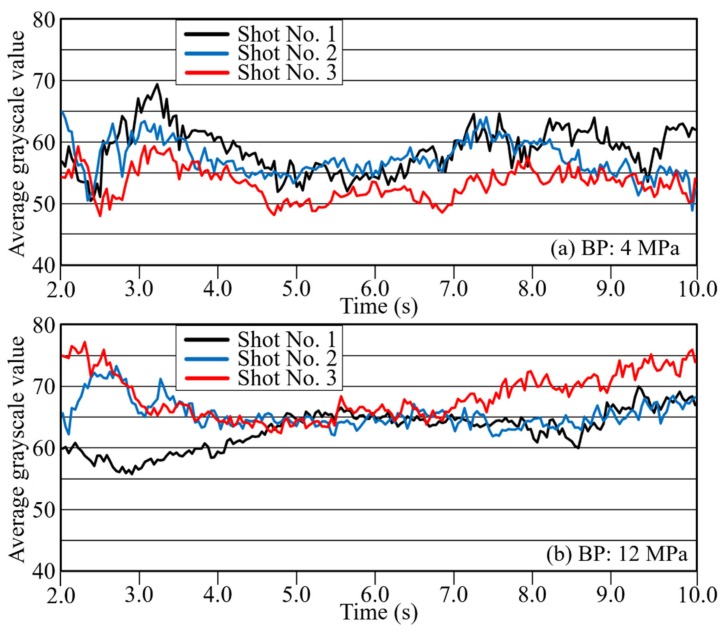
Change curves with time of average grayscale under a back pressure (BP) of (**a**) 4 MPa and (**b**) 12 MPa (glass fiber reinforced polypropylene (PP)).

During measurement of the reciprocating-screw plastication process at each back pressure, the screw position receded linearly with time, with a metering time of 14.4 s for a back pressure (BP) of 4 MPa, 15.72 s for BP 8 MPa, and 17.02 s for BP 12 MPa. To compare typical injection processes, Figure 4 shows the changes in average grayscale *I*_ave_ with time during the injection process of the metering resin at BPs of 4 MPa and 12 MPa for three shots each. The observed changes in the average grayscale *I*_ave_ with time were interpreted as changes in fiber distribution in the molten resin since the undefibrated areas and areas of non-uniform fiber distribution became dark due to poor light transmission. Figure 4 shows that, with increasing BP, the average grayscale *I*_ave_ during flow became more consistent, while the fiber distributive uniformity improved. In particular, at a BP of 12 MPa, average grayscale became almost fully consistent across shots in the middle of the flow (ca. 4 to 7 s). In addition, the overall grayscale level was slightly higher than at a BP of 4 MPa. It is believed that high BPs encourage fiber defibration and distributive uniformity by means of high resin pressure and long mixing time (metering time).

#### 3.1.2. Evaluation of Pilling Area

**Figure 5:**
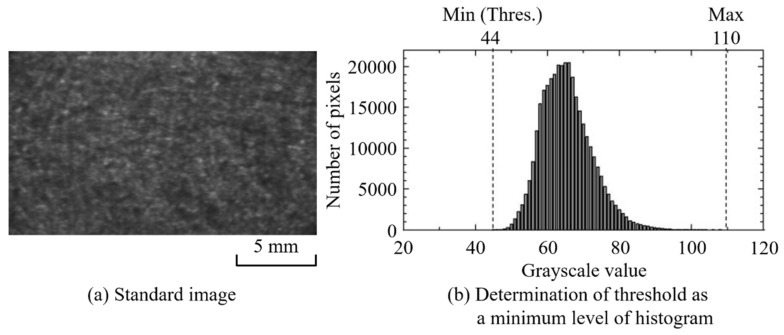
Standard image to determine the value of the threshold (glass fiber reinforced PP; BP = 12 MPa, Shot No. 1).

**Figure 6:**
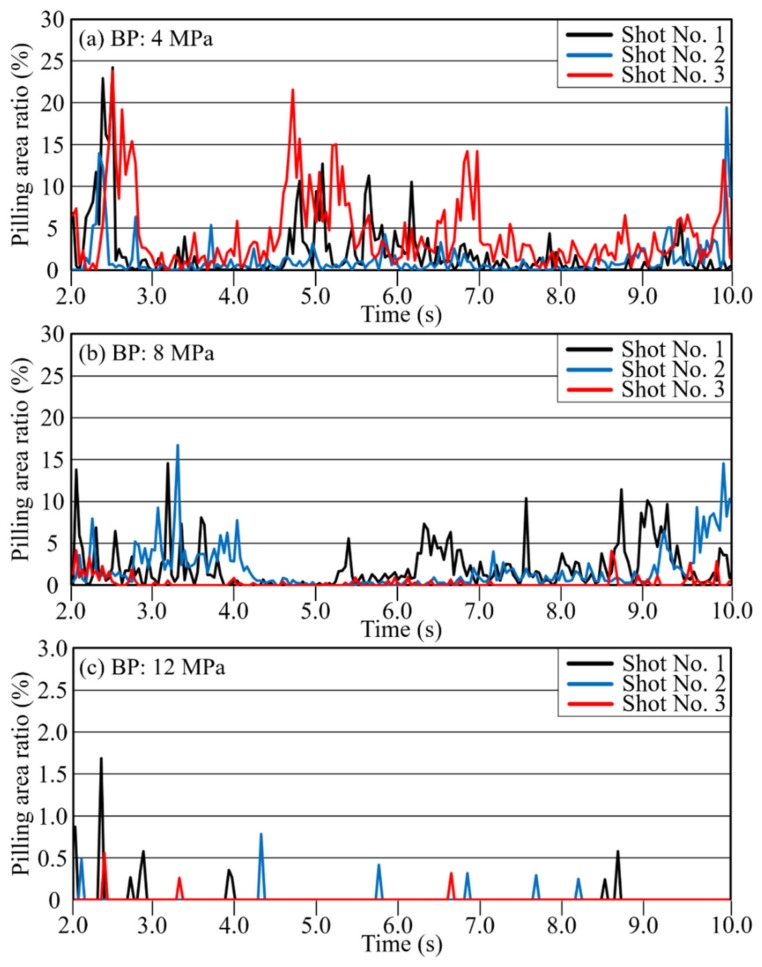
Change curves with time of pilling area ratio under BP of (**a**) 4 MPa, (**b**) 8 MPa, and (**c**) 12 MPa (glass fiber reinforced PP).

**Figure 7:**
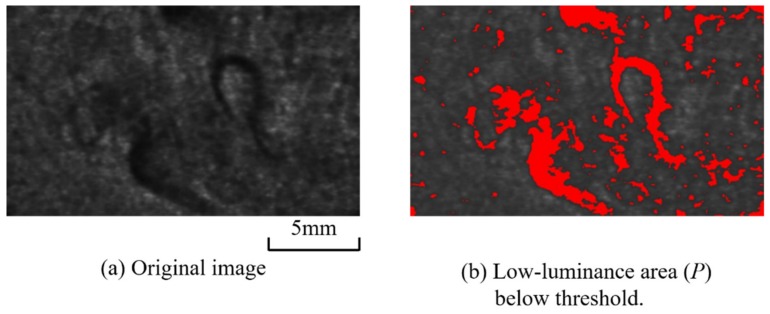
Calculation procedures of pilling area *P* (BP = 4 MPa, Shot No. 1, 6.12 s).

The threshold value *Thres* in formula (1) can be set arbitrarily, depending on the extraction sensitivity of the grayscale unevenness to be evaluated. In the present study, the image having the most uniform grayscale among all photographed images was selected as the standard image, and the minimum value obtained from that image’s grayscale histogram was set as the threshold *Thres*. Figure 5 shows a threshold-setting example. The minimum grayscale value of 44 was set as *Thres*, and the dark area *P* was counted. Figure 6 shows the time variation of the pilling rate α calculated according to this threshold. Table 2 shows the pilling rate averaged over 10 seconds for each injection period. The higher the BP, the smaller the pilling rate. The pilling rate at 12 MPa was 3% of that at 4 MPa and 5% of that at 8 MPa. In addition, the variation between shots was reduced. At a BP of 4 MPa, *P* showed substantial variation across points during injection, but at BPs of 8 MPa and 12 MPa, *P* was elevated only at the injection sequence’s beginning and end. This behavior is interpreted as promotion of defibration and distributive uniformity in the middle of the plastication process, preceded and followed by a temporarily lower fiber length distributive uniformity during the long-fiber-reinforced resin’s reciprocating-screw plastication process [19]. Figure 7 shows an example of the evaluation image, (a) showing the original image and (b) showing the extracted dark area. In this dark area, clear fiber bundles and small irregularities with poor distribution are counted as pilling areas. The fiber bundles’ occupation of a relatively large area suggests that both can be classified further according to the extracted dark area’s size.

### 3.2. Bright Area Analysis Method

In cases where the long carbon fiber concentration is high, the molten resin becomes opaque when the fiber content is increased as the fiber bundle is defibrated and dispersed. Therefore, when the backlight transmittance decreases, the grayscale histogram becomes unevenly distributed in the dark area, making it difficult to extract the pilling area from the dark area in the image. On the other hand, the bright area is generally distributed over a wider range than the dark area. When the undefibrated fiber bundle area begins to defibrate and the fiber disperses further, the light transmittance of the initial molten resin in the areas between the undefibrated fiber bundles gradually decreases from the transparent state. As a result, the brightness of the bright area in the image decreases and becomes opaque (dark area). The light transmittance in the image is interpreted as an indicator of the degree of undefibration and distributive non-uniformity. Here, we propose a bright area analysis method for evaluating distributive uniformity by observing wide bright areas.

The extraction procedure for the bright area is basically the same as the extraction procedure for the dark area (pilling area) in Section 3.1. For the dark area analysis method, a darker area whose grayscale threshold is lower than the set value is extracted. Contrariwise, the bright area analysis method extracts pixels brighter than, or equal to, the grayscale threshold’s set value. For this reason, we attempted to evaluate defibration and distributive uniformity of 10 wt % long carbon fiber content in the bright area forming a wide grayscale distribution, in order to ensure a certain level of light transmittance by the backlight. That is, we extracted the distribution on the higher grayscale side than the peak value of the histogram. The sampling interval of the images was set to 20 ms so that the moving distance based on the maximum flow velocity *V*_max_ was equivalent to the flow length of 9.7 mm in the evaluation area. The evaluation area *S* in this experiment was 480 × 420 px.

#### 3.2.1. Time Variation of Average Grayscale during Resin Flow and Evaluation of Pilling Area

**Figure 8:**
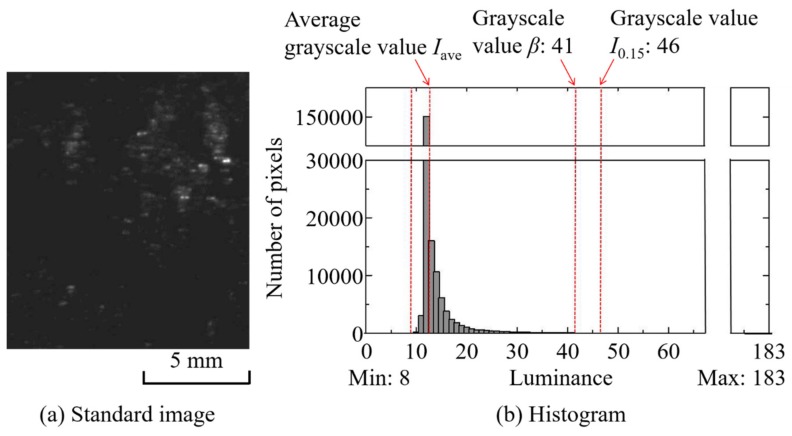
Standard image for determining the threshold value (CFRP); BP = 12 MPa, Shot No. 1.

**Figure 9:**
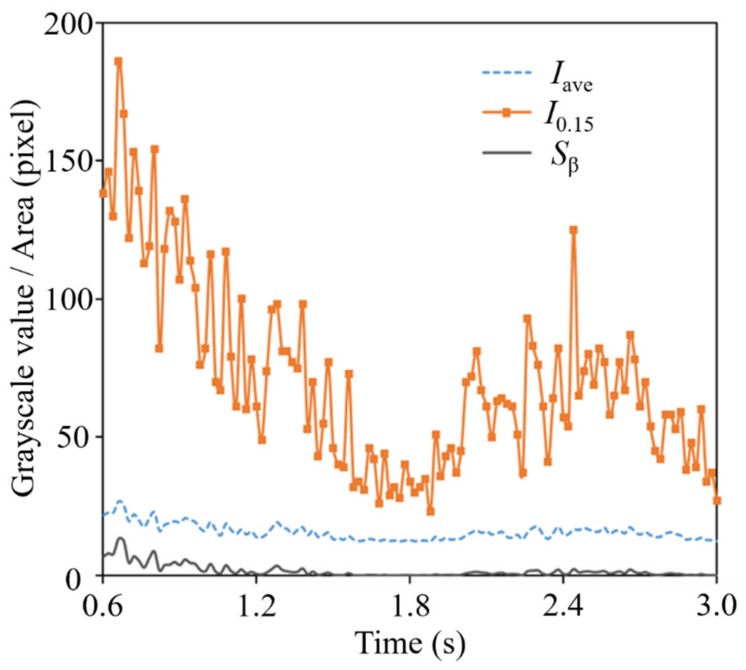
Changes of carbon fiber reinforced polymer in each of the three indicators with time. ((1) Pixel brightness *I*_0.15_ corresponding to the top 0.15% of pixels ordered by brightness; (2) area (number of pixels) *S*_β_ above the brightness *β* of the image; (3) average brightness *I*_ave_ of the image.)

Figure 8 shows (a) a standard image and (b) a histogram of standard images (Shot No. 1, 1.96 s) for carbon fiber content of 10 wt % and a BP of 12 MPa. Based on the measurement image of the same shot, the following time variations were obtained and are compared in Figure 9: (1) pixel brightness *I*_0.15_ corresponding to the top 0.15% of pixels ordered by brightness, (2) area (number of pixels) *S_β_* above the brightness *β* of the image, and (3) average brightness *I*_ave_ of the image. *β* is an arbitrary value between the overall average grayscale value in the entire captured image and the average value of grayscale *I*_0.15_ in the entire captured image. *I*_0.15_, which is intended to correlate mainly with distributive uniformity, can be regarded as representative of the highest transparency (i.e., areas with poor distribution) among the resins that fill the areas between the residual fiber bundles. This was set to 0.15% to reduce image sensor noise. *S_β_* is a large area spot and is intended to correlate mainly with poor defibration. As *I*_0.15_ is brighter, the non-uniform area of *S_β_* spreads uniformly, and a positive correlation is expected between them. In addition, as shown in Figure 9, the changing trends as well as its relevancy in *I*_0.15_, *S_β_*, and *I*_ave_ are relatively close suggesting that average grayscale *I*_ave_ can be used simply as an index for defibration and distributive uniformity evaluation. In the case of 10 wt % long-carbon-fiber resin, the average grayscale *I*_ave_ was 13, and *I*_0.15_ was 46. *β* was set to 41, an arbitrary value between the average value of all images’ average grayscales *I*_ave_ and the average value of all images’ *I*_0.15_ values. In order to evaluate defibration and distributive uniformity, *I*_ave_ and *S_β_* were measured, and a pilling rate *α* was obtained. Long-carbon-fiber-reinforced resins generally have a carbon content of 15 wt % or more, but when the carbon fiber content was 15 wt % or more, the light transmittance required for analysis could not be realized. To increase the light transmittance, it is necessary to reduce the channel thickness, increase the backlight source’s brightness, and increase the photographic camera’s sensitivity. Therefore, we conducted a visualization experiment at a content rate of 10 wt % to establish an analytical method to evaluate fiber distributive uniformity.

#### 3.2.2. Effect of Pellet Input Method

**Figure 10:**
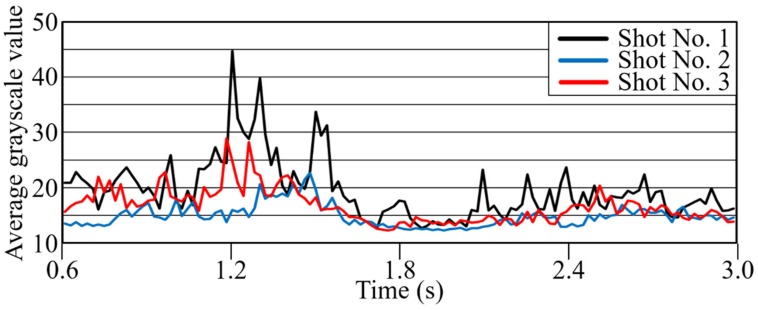
Average grayscale change curves with time of 1000 g dry-blended pellets into a hopper (BP = 8 MPa).

**Figure 11:**
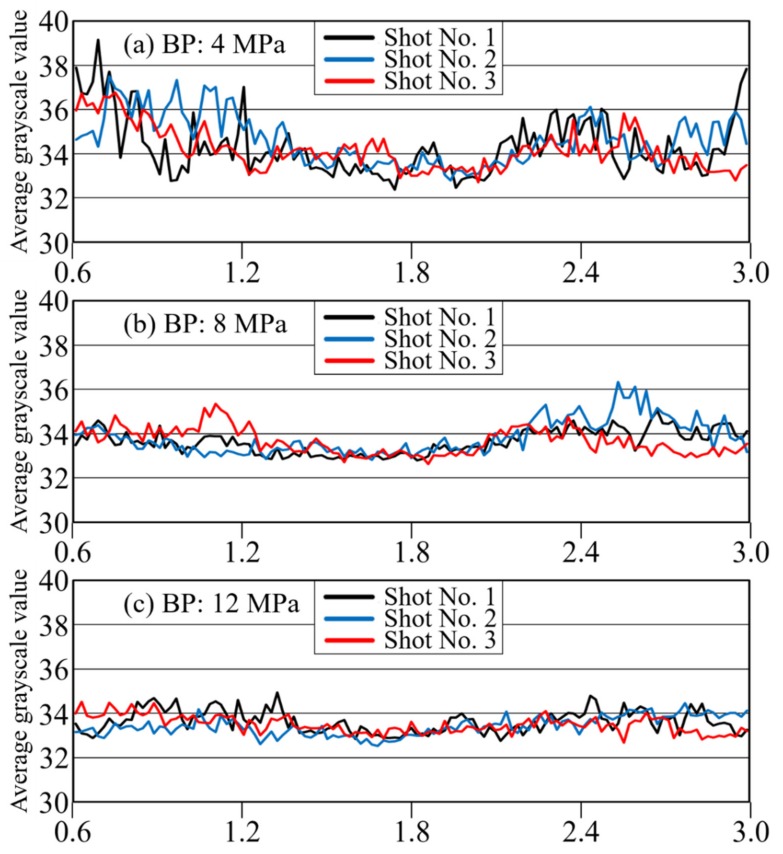
Change curves with time of average grayscale under BP of (**a**) 4 MPa, (**b**) 8 MPa, and (**c**) 12 MPa (CFRP, 10 wt %).

**Figure 12:**
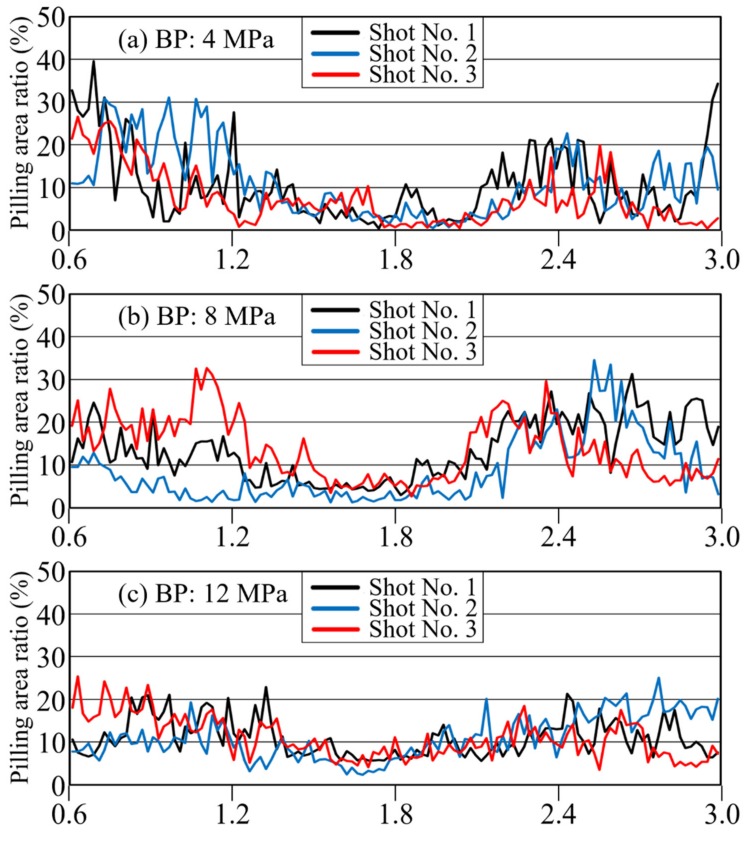
Change curves with time of pilling area ratio under BP of (**a**) 4 MPa, (**b**) 8 MPa, and (**c**) 12 MPa (CFRP).

In this experiment, the long carbon fiber content was adjusted by dry-blending PP at a predetermined ratio into high-concentration 20-wt % carbon fiber original pellets for dilution. This dilution method is commonly used in production sites. This dry-blending operation is performed in an off-line batch process, and then the dry-blended material is placed directly in the hopper. Figure 10 shows the average brightness changes with time when blended pellet materials from a batch process of 1000 g were placed in the hopper continuously and molded when the hopper was almost empty. Low reproducibility between three consecutive shots was confirmed. The causal factors of these phenomena were interpreted as follows. In the dry-blending process, the pellet shape differed significantly between the elongated shape of long-carbon-fiber pellets (length ca. 6.7 mm) and the round shape of “rice-grain” PP for dilution prepared by hot cutting. The long-carbon-fiber pellets experienced substantial friction along the wall surface on the inner wall of the hopper, on the conical surface of the deep part of the hopper, and on the surface of the resin pellets supplied to the heating cylinder. For this reason, compared to the round PP pellets, the moving and falling speed of the long-carbon-fiber pellets gradually slowed, and the long-carbon-fiber pellets tended to stay along the wall surface. In this framework, when there was less material in the hopper, the influence of the wall surface on the fiber pellets became more obvious, the long-carbon-fiber pellet content dropped into the cylinder increased, and the material mixing ratio became non-uniform. In this measurement experiment, in order to reduce the influence of defibration and distributive uniformity of carbon fiber pellets, the batch size was reduced to 500 g, and the material in the hopper was completely removed every three consecutive shots. There was no variation under these conditions, as shown in Figure 10 (see also Figure 11 and Figure 12). The unstable phenomenon above is interpreted as generated by the dry-blending of pellet materials of different shapes and sizes; we demonstrated that the phenomenon could be evaluated quantitatively using a mixed-evaluation mold. To avoid this phenomenon, measurement experiments were performed on 500 g batches of pellets with complete replacement of pellets in the hopper.

#### 3.2.3. Effect of BP on Fiber Distribution

Figure 11 and Figure 12 show the average grayscale *I*_ave_ and the pilling rate over time for each of the three back-pressure conditions, with 3 shots included for each condition. These results confirm that the three shots can be reproduced well. As defibration and distribution effects were generated, the overall time variation of the grayscale value also increased. When the distribution became uniform, the transmitted light in the area became uniform, and the overall brightness decreased. The results shown in Figure 11 and Figure 12 confirm that the pilling rate decreased with increasing BP. Reproducibility was extremely high, especially in the middle of the injection process at a BP of 12 MPa. As confirmed for long-glass-fiber resin, the metering time was longer for higher BPs, and the mixing time was higher as well due to the shearing action. This behavior confirms that defibration and distributive uniformity are improved greatly at higher BPs. In addition, the brightness gradually decreased from the start of injection and then increased again at the end of injection. As stated in Section 3.1.2, this phenomenon corresponds to the phenomenon [19] in which the fiber length distribution of long-glass-fiber resin decreases temporarily in the middle of the plastication process’ metering. These results confirm that high BP promoted distributive uniformity while reducing average brightness.

### 3.3. Comparison Between Dark Area Analysis Method and Bright Area Analysis Method

We evaluated the possible equivalence between the dark area evaluation method (for glass fiber and low-concentration carbon fiber) and the bright area evaluation method (for high-concentration carbon fiber). In order to clarify the correlation between the two methods, an observation experiment was conducted on 5 wt % long carbon fiber as an evaluation target, at a rotation speed of 60 rpm, in a well-balanced condition for which the histogram formed bright areas and dark areas (i.e., areas below and above the histogram’s peak value).

**Figure 13:**
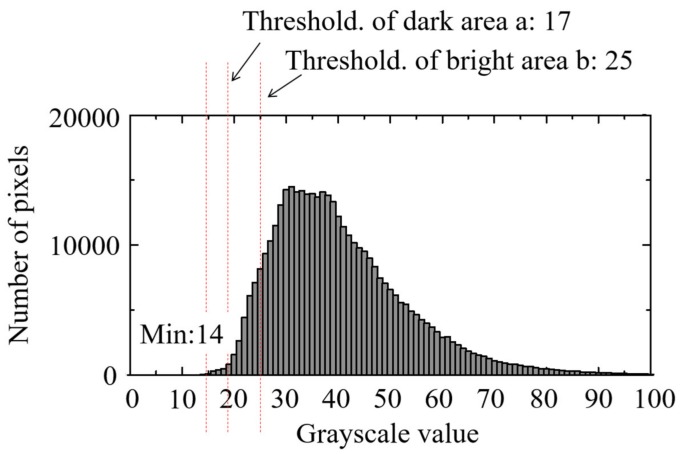
Histogram of standard image (CFRP 5 wt %, BP = 4 MPa, 60 rpm, Shot No. 2).

**Figure 14:**
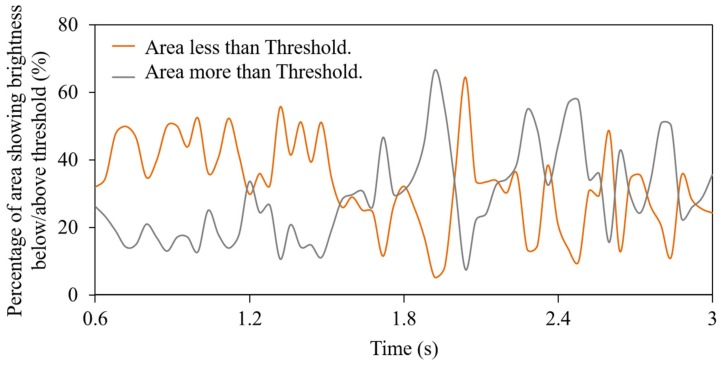
Comparison of the average brightness change curves with time, calculated based on dark area and bright area (CFRP 5 wt %, BP = 4 MPa, 60 rpm, Shot No. 2).

Figure 13 shows the histogram of the standard image. The results of extracting the dark area and bright area are shown in Figure 14. As shown in Figure 13, threshold *a* was set in the wide distribution in the histogram’s bright area and *b* in the slightly narrow distribution in the dark area. Here, the bright area extraction threshold *a* was 25, and the dark area extraction threshold *b* was 17. These two threshold values were selected based on the grayscale difference of 4 between the global average grayscale of 21 in the measured images during the time interval from 0.6 s to 3 s. For individual captured images, the area *A* of the bright region with brightness values above threshold *a* and the area *B* of the dark region with brightness values below threshold *b* are complementary. Therefore, when changes in both with time are superimposed, as shown in Figure 14, the relationship is always antiphase. When the pilling rate was calculated based on Figure 13, the pilling rates of images obtained from areas *A* and *B* were not necessarily the same. Areas with large fluctuations in the bright region were slightly out of phase, but there were also large fluctuations in the dark region. In general, it was possible to detect an area with a high pilling rate (area with significant undefibration) for each method. This means that, when the total volume of carbon fibers in the resin flowing in the nozzle was constant, and the resin flowing in the nozzle was removed within a short period of time, while the field of view of the entire area became dark (bright), an area with a bright (dark) field of view appeared conversely to compensate for it. Therefore, an extremely bright area could easily pass by after an extremely dark area passed. As described above, it is assumed that the same evaluation result could be obtained from the bright area or from the dark area given sufficiently large overall amplitudes. For carbon fiber content exceeding 5 wt %, the field of view became considerably dark, and the dark area *B* on the histogram had a very narrow distribution width. Since the calculation based on the dark area *B* was substantially difficult, inevitably an evaluation based on bright area *A* needed to be used. The method depended on whether the grayscale distribution of the dark area *B* had a width that could sufficiently distinguish levels of grayscale.

## 4. Conclusions

(1) In order to evaluate the defibration and distributive uniformity of long fiber bundles inside the fluid resin injected via the nozzle, a defibration and distributive uniformity evaluation mold was developed. This mold was able to show resin passing through a thin channel between opposed parallel glass blocks, observable by backlight. Evaluation experiments using 30 wt % long-glass-fiber-reinforced PP and 10 wt % long-carbon-fiber-reinforced PP empirically clarified that this method was effective for evaluating defibration and distributive uniformity.
(2) Image analysis extracted undefibrated and non-uniformly dispersed fiber bundle areas as dark areas in the backlight image, classifying the area as a pilling area. In this way, a dark area analysis method was proposed based on the time variation of quantified changes in pilling rate. This method was applied to the analysis of a plastication process of 30 wt % long-glass-fiber-reinforced PP, by changing the BP in the plastication conditions, clarifying that pilling rates and distributive uniformity were both improved at high BPs. Furthermore, the pilling rate became higher at the beginning and end of injection. This finding suggests a fiber length change correlated with defibration and/or distributive uniformity.
(3) Opaque fibers and low-brightness backlight images make it difficult to extract the pilling area from the dark area. Therefore, we proposed an evaluation method using the maximum grayscale in the bright region as the evaluation index for distributive uniformity and the spread (area) of the bright region as the evaluation index for defibration. These indices were applied to the analysis of 10 wt % long-carbon-fiber-reinforced PP, confirming that, for high-density, long-carbon-fiber pellets dry-blended with natural PP, concentration fluctuations occur easily due to re-separation in the hopper. We also confirmed that high BP promotes defibration and distributive uniformity of the fiber bundle by changing the BP in the plastication conditions. Finally, we showed that the brightness decreased while the pilling rate decreased in the middle of the metering process.
(4) Equivalence between the dark area analysis method and the bright area analysis method was evaluated using 5 wt % long-carbon-fiber-reinforced PP. In both analysis results, the image’s average grayscale fluctuation showed an antiphase correlation. Though the absolute value of the pilling rate fluctuation was not necessarily identical for the two methods due to dependence on the set threshold values, similar evaluation results were obtained from the dark area and from the bright area.

Our evaluation mold is a device with high applicability at the molding site because it can be mounted on a wide variety of injection molding machines. The device can be used to evaluate the distributive uniformity of highly transparent reinforcing materials and fillers such as long glass fibers in a variety of contexts. On the other hand, a completely opaque reinforcing material such as carbon fiber can hardly transmit backlight, depending on the fiber content. For this reason, the application range of the evaluation mold developed in this study is generally up to 15 wt % for long carbon fiber. Thus, at this stage, it is difficult to evaluate the dispersibility of the fibers using a carbon fiber reinforced resin of 15 wt % or more. To expand the application range to more than 20 wt %, improvements must be made such as reduced channel thickness from the current 1 mm. It is considered that the amount of backlight transmitted through the gap between the fibers is increased, and the fiber defibration and distributive uniformity can be evaluated using the bright area analysis method. Further, improvements such as increasing the backlight intensity will be required.

## Figures and Tables

**Table 1:** Molding conditions.

Item	Setup Value
Cylinder temperature [°C] Nozzle/Cylinder 1/Cylinder 2/Cylinder 3/ Cylinder 4/Hopper	220/220/220/215/210/50
Mold temperature [°C]	217 (Measured value)
Rotation speed [rpm]	120
Back pressure [MPa]	4/8/12
Screw stroke [mm]	30 (carbon fiber reinforced polymer, CFRP)/100 (glass fiber reinforced polymer, GFRP)
Screw injection speed [mm/s]	10

**Table 2:** Change of pilling rate with elapsed time.

Back Pressure	Ten-Point Average Pilling Rate (%)			
	Time Zone			Average
	2.0–4.48 s	4.52–7.48 s	7.52–10.0 s	
4 MPa	14.81	13.07	9.21	12.36
8 MPa	9.06	5.12	10.49	8.22
12 MPa	0.538	0.271	0.305	0.371
